# Supplementary material for: Fate of Entosis: From the Beginning to the End in Untreated Advanced Breast Cancer
Source: Int J Mol Sci. 2023 Jul 29;24(15):12142. doi: 10.3390/ijms241512142 (PMC10418304; doi:10.3390/ijms241512142)
Supplement: Supplementary file 1 [file ijms-24-12142-s001.zip › ijms-2499082-SI.pdf]

Supplementary data.

S1. Macroscopic image description and Microscopic preparations

S1.1. The study on T/N; Macroscopic image analysis: An unmarked right mammary gland, with the contents of the axillary fossa (measuring 24 x 22 x 5.5 cm; 1768 g) covered with an ellipse of skin (21 x 18 cm), was obtained. An ulcerated bump (10.5 x 7.5 x 5.0 cm), highlighting and infiltrating the skin was identified. Macroscopic margins were as follows: upper margin of 2.5 cm, lower margin of 11.0 cm, a margin on the chest side of 1.5 cm, a margin from the armpit side of 9.0 cm, and a margin from the middle of 6.0 cm. Inside the axillary cavity, a fat-shaped package of axillary lymph nodes measuring 7.5 x 5.5 x 3.5 cm was identified. On the surface of the skin, an ulcer (with a diameter of 1 cm) was found.

Microscopic preparations (T/N) were composed of:
1) Tumor with skin
2) Tumor with margin from the chest side
3) Tumor from the central part
4) Other parts of the tumor
5) Lower quadrants outside the tumor
6) Upper quadrants outside the tumor
7) Lymph node package starting from the top
8) Nipple from the side of the tumor

S1.2. The study on relapse (M); Macroscopic analysis: A mammary gland (28.5 x 23.0 x 8.00 cm; 1729.0 g) and the contents of the axillary fossa (12.5 x 5.0 x 3.0 cm) were obtained for the study. On the cross-sections, the adipose fibrous gland, without any obvious tumors, was identified. In the inferior medial quadrant, fibrous tissue formed two star-shaped structures. In the inferior outer quadrant, irregular, whitish lesions (3.5 x 3.0 cm) and puffs of whitish tissues closer to the armpit were observed. In the armpit itself, two saddle-shaped well-demarcated tumors measuring 3.5 x 3.0 x 3.5 cm and 2.0 x 1.5 x 2.5 cm were found. Individual quadrants were examined serially by taking numerous sections and preparing microscopic slides.

The following microscopic preparations were prepared from the relapse (M):
1) Inferior medial quadrant
2) Inferior outer quadrant
3) Upper medial quadrant
4) Upper outer quadrant
5) Borders of the outer quadrants
6) Subpapillary region
7) Nipple
8) Fatty tumors of the armpit
9) Other lymph nodes of the axillary fossa
10) Separately lying skin fragment with infiltration

S2. Suplementary Figures: The whole slides images.

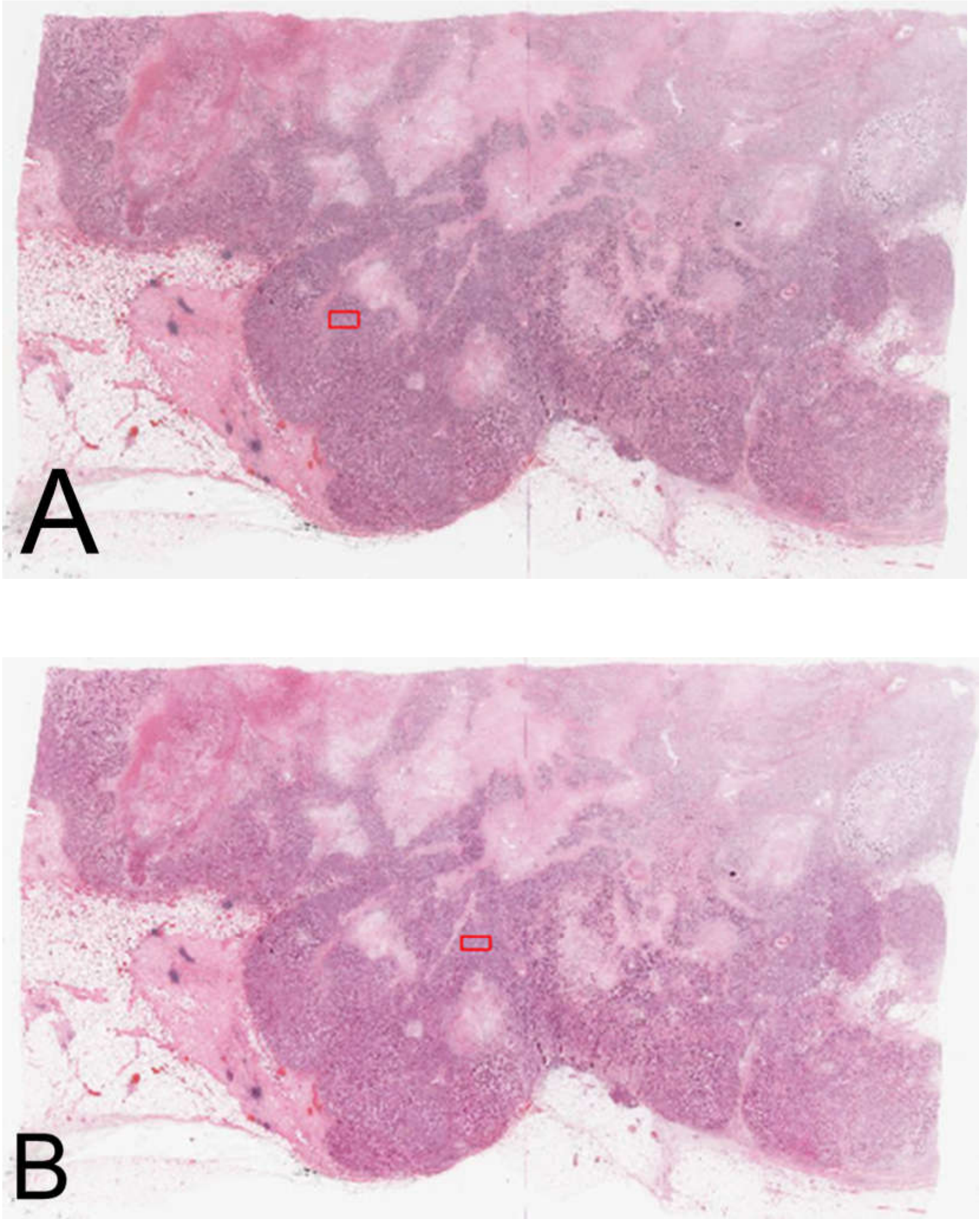

Figure S1. The whole slides images H&E staining of the primary cancer lesion (T). Entotic hotspots marked using red rectangles corresponding to Fig. 2 A,B.

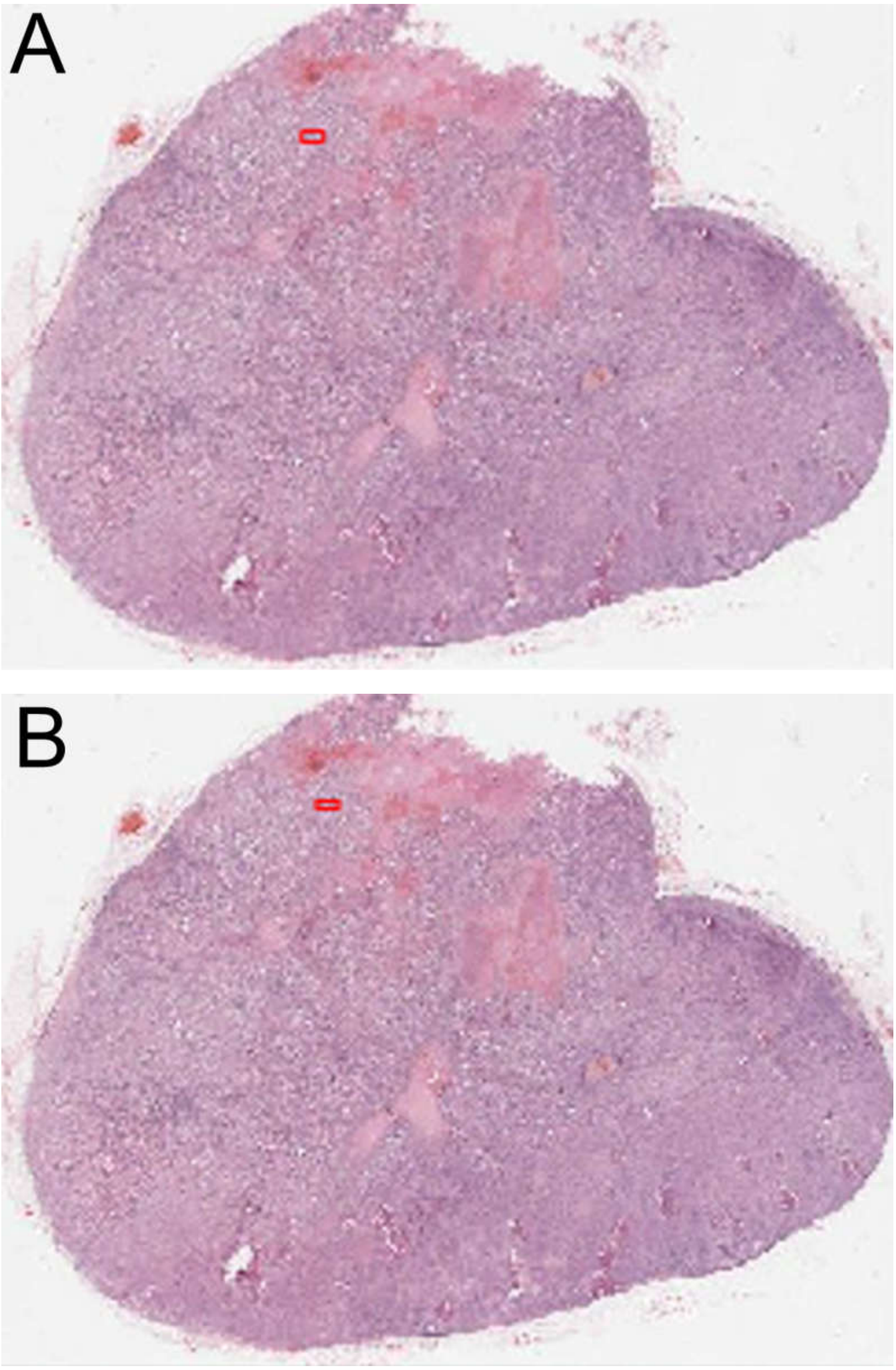
A
B

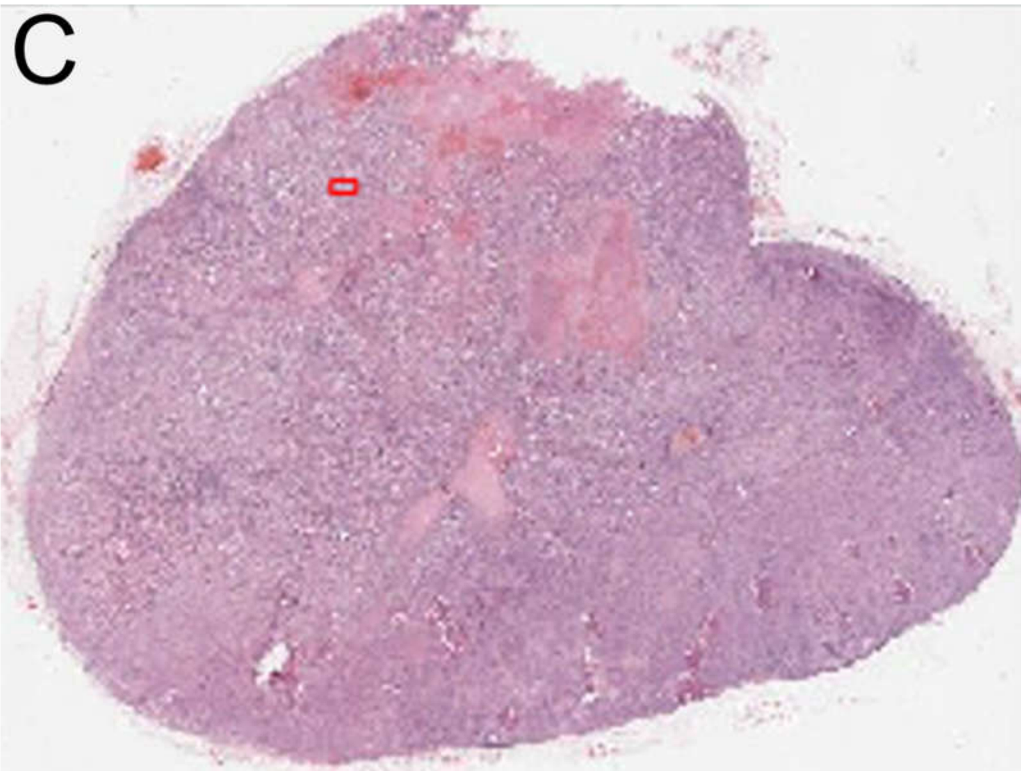

Figure S2. The whole slides images H&E staining of H&E staining of the lymph node metastasis (N). Entotic hotspots marked using red rectangles corresponding to Fig. 3 A,B,C. Scale bar 2 mm.

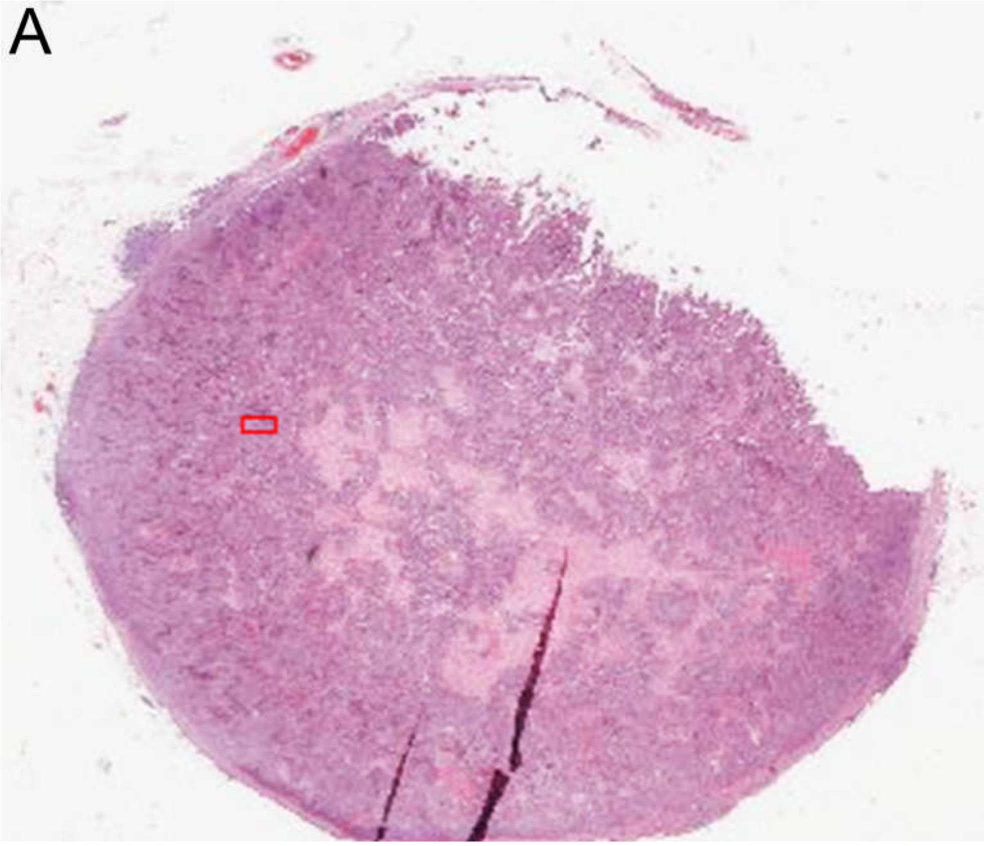

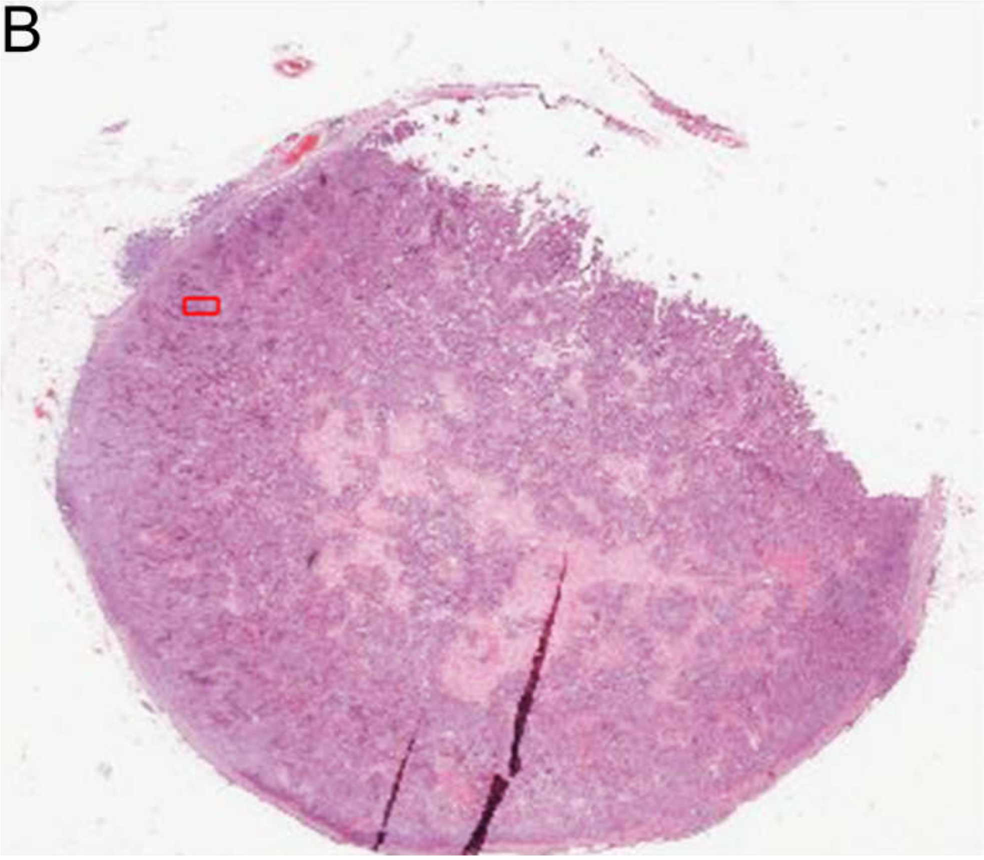

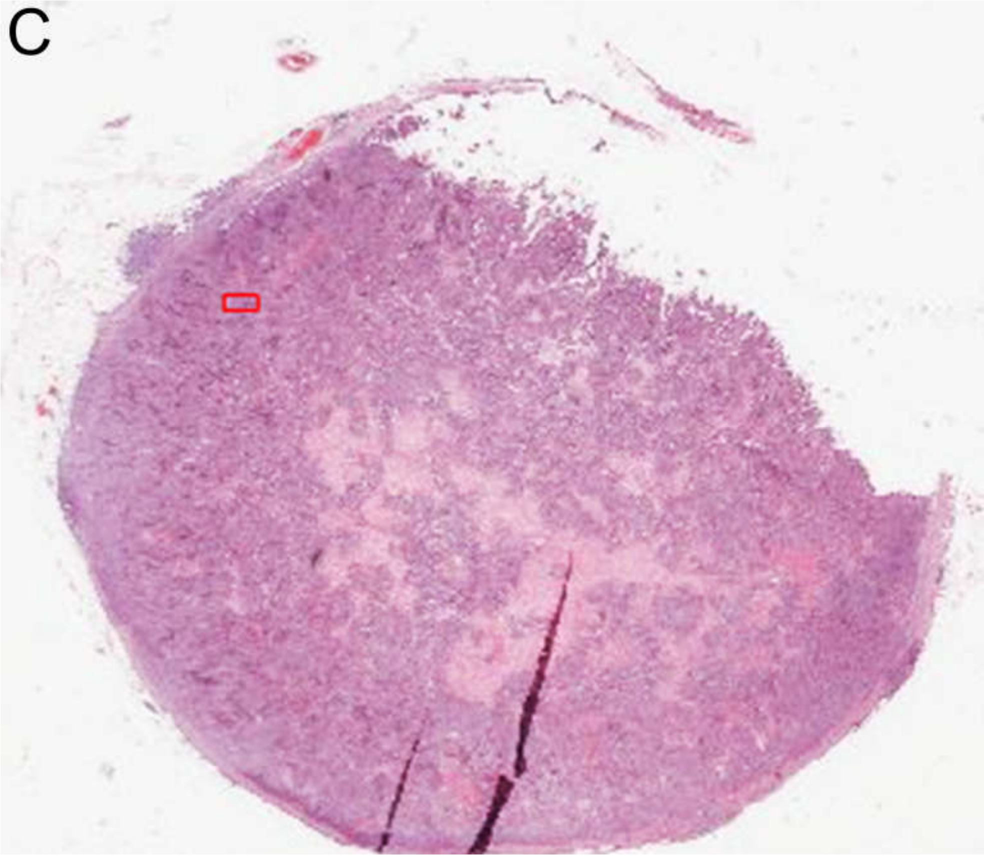

Figure S3. The whole slides images H&E staining of the contralateral axillary lymph node far metastasis (M). Entotic hotspots marked using red rectangles corresponding to Fig. 4 A,B,C.
